# Hamlet’s Insanity

**Published:** 1873-09

**Authors:** Horatio R. Bigelow

**Affiliations:** Boston, Mass.


### Chicago Medical Journal.

A MONTHLY RECORD OF

*Medicine, Surgery and the Collateral Sciences.*

EDITED BY J. ADAMS ALLEN, M.D., LL.D.; AND WALTER HAY, M.D.

VOL. XXX. — SEPTEMBER, 1873. — NO. 9.

### Original Communications.

ARTICLE I.—*Hamlet's Insanity.* By HORATIO R. BIGELOW, M.D., Boston, Mass.

Were it not for the fact of its recent revival, it would seem to be scarcely necessary to revivify the old discussion which modern inquiry had so satisfactorily laid to rest. In his recent lecture in New York City, Mr. Mac Donald not only emphatically asserted his conviction of Hamlet's sanity, but even ventured the statement that no student of the present day believed otherwise.

With this latter we have little to do, for he who has followed the scientific inquiry with which the past decade has been rich and rife, can easily confute so rash and venturesome a tale, by citing the opinions of pathologists, psychologists and physiologists of acknowledged ability. Strange too, it is, that in the face of evidence and fact, Mr. Mac Donald should have thus committed himself; for since the occasion was a public one, his words become legitimate subjects of criticism. The facts which were adduced to establish the sanity of Hamlet, were so weak, so illogically put together, and so entirely contradictory, as to militate against the celebrity of the lecturer, rather than add to his trans-Atlantic reputation.

Before coming to the ultimate discussion, it will be necessary, by way of preface, to form some definite ideas upon the following points:

1. What mental condition is it, which entitles any one to judge of the creations of genius or to read the secrets of the minds of others?
2. What is insanity?
3. How may it be caused?
4. What are its divisions?
5. Was the creation under observation—Hamlet—insane?

The necessarily narrow limits to which journal articles are restricted will be one excuse for the brief manner in which each of these divisions will be treated; but if some new field of thought be opened up to the unbiased student, one object will be attained. The extent of our adaptation to external realities by prototypical cerebral reaction will be the measure of our mental astuteness; the more harmonious the creation and outward action of our ideas, the more fitly adapted are we to peer into the ideational reactions of others. The mere metaphysical, subjective introspect will avail us to no purpose; for the abstraction which is necessary for the performance of conscious scrutiny, withdraws from mental freedom, that which is necessary for its perfected entity, and creates abnormal existence in the object to be analyzed by self-consciousness. Thus, such a method, by rendering our own mental state unhealthy, is entirely incapacitated in its application to others. One idea must follow another in logical sequence, insensibly blending one with the other, as the wave motion of the wind travels over a field of grain, its residual force being transferred from cell to cell, creating in each its proper function. The force generated by wholesome thought is partially expended outwards, the residue furnishing the stimuli of future creation. If an idea fail to act in this manner, there ensues a prolonged tension of the ideational centre, and self-consciousness results. It is not by any subjective analysis of our own minds that we can correctly judge of others, but rather from a consideration of man's relationship to external objects; from the study of the higher faculties in the animal, as well as in all the phases of the human kingdom, the babe, the insane, the idiot, and from the knowledge which we thus by acquisition attain, of the results which certain stimuli in the ideational cells bring about. That which we would do under similar surroundings, is no criterion of the action of others. Will is not an innate, constant quantity, but a variable force, arising from the experience of past generations, determining an event, but in no wise governing the means by which the end is consummated. Forgetting, so far as is possible, our consciousness in thought, (for when we once become conscious that we are thinking, an unhealthy action results), we must study the mind in all its varied phases of existence and action, and in this way only can we become polished critics.

2. What is insanity?

In reply to this question, we will give the definitions of two of the ablest thinkers, in the particular field of mental diseases, which our age has produced. Dr. Maudsley, of London, thus defines it: "A morbid derangement, generally chronic, of the supreme cerebral centres—the gray matter of the cerebral convolutions, or the intellectorium commune, giving rise to perverted freedom, defective or erroneous ideation, and discordant conduct conjointly or separately; and more or less incapacitating the individual for his due social relations."

Dr. Hammond, of New York, says: "A manifestation of disease of the brain by general or partial derangement of one or more faculties of the mind, and in which, while consciousness is not abolished, mental freedom is perverted, weakened or destroyed."

Mind then, it is presupposed, is the perfected force generated by the supreme or ideational centres, represented in the hemispherical convolutions and cortical cells of the brain. When these cells cease to react upon each other harmoniously, when an idea prolongs its tension so as to tyrannize over the understanding and become an absorbing entity, illusions and delusions result, and the person is pronounced insane. When the sensory ganglia are abnormally active or viciously constituted, their reflex and transmitted actions become disordered; we have, as a direct cause, hysteria, and indirectly, through the force transmitted upward, a phase of insanity.

It is important to remember, that mind is not an abstract, incomprehensible agent, but that it is a natural force, and that its location is the brain; that to call it into action, certain stimuli alone are needed. With this consideration fresh in our memory, we can better understand the chief point.

3. What are the causes of insanity?

Naturally, anything that will interfere with the healthy action of the mental function—inherited acquisition of vicious action; want of vitality in the nervous tissues; improper cerebral nutrition; association; shocks; excessive emotional activity; and various other causes which it would be superfluous to narrate. By far the most frequent cause, is the inherited tendency. The various neuroses, which, in every generation, and in members of the same generation, will often assume a variety of forms, will engender the peculiar abnormal psychological condition, which a sudden shock will develop into true insanity. The circumstances of life may imbue the mind with an idea so absolute, as to render the mental action secondary; the idea becomes morbidly conscious, it dwells constantly upon itself, and rears up the various unhealthy mental phenomena. Our associations may be such as to induce it. Many periods in the world's history have been characterized by such tyranny of action. The dancing epidemics, and the superstitions of every age, are samples. Constant communion with those imbued with such creeds, will taint, oftentimes, the healthiest mind. Religious fanaticism, excess of religious observance—the one a result of insanity, and the other a cause—have marked each step in the world's advance. Hamlet himself explains its cause, when he is made to say—

> "So, oft it chances in particular men,
> That for some vicious mole of nature in them,
> As, in their birth, (wherein they are not guilty,
> Since nature cannot choose his origin,)
> By their o'ergrowth of some complexion,
> Oft breaking down the pales and forts of reason." *

It may result from the emotional condition. Spinoza has divided the emotions into three classes: 1. Pain; 2. Joy; 3. Desire. A morbid activity in any of the ganglia representing these conditions may transmit its poisoned tone upward, and upset the reason. Visceral disease may cause a variety in this way: a sense of the existing trouble is transmitted through the proper channels to the sensory ganglia; these become unduly active, and send upward false impressions to the brain. The idea, thus excited, becomes dominant, directing its sole attention to the affected organ, exaggerating

* Act 1, Scene 4.

its defect, creating an infinity of ailments, and we have one form of hypochondria.

4. What are its divisions?

It is sufficient for our present purposes, to adopt the usually received division of Esquirol:

Mania, { Acute. Chronic. Recurrent.

Monomania.

Melancholia.

Moral Insanity.

Dementia, { Primary. Secondary.

Idiocy.

General Paralysis.

It will not be necessary to dwell at length upon this head, or upon the localization of the various lesions; a proper consideration will enable us without difficulty to recognize the different mental departments or divisions involved in each.

5. If we have considered carefully the nature and causes of insanity and the regular working of the human mind, much of seeming difficulty will be wiped away from the discussion of the final question. We can also, now, look more closely into Mr. Mac Donald's statements, and apply, as corroborative proof of Hamlet's insanity, the very facts which that lecturer adduced to maintain his own position—that of Hamlet's mental sanity. And first, what was the condition of the continent of Europe, in regard to its habits of thought, during the time of Hamlet's life, and for a period of years before that time? Every decade of that eventful century was marked by some peculiar general aberration of mind, which, commencing in a remote corner, rapidly spread its flight throughout the land, gathering in its ways old and young, rich and poor. The superstition of religion, the abstraction incident upon the peculiar studies so popular at that time, that peculiar psychological condition which inaugurated the orgies of St. Jean Baptiste in France, the dancing mania of Italy and in Germany, were the excrescences bursting out of the accumulated experience of generations, and which were generated by unhealthy mental condition. It was an age peculiarly fitted for the advancement of the most paradoxical theories, for the development of unnatural habits of reverie, well suited to foster long-continued and abnormal mental strain, directed toward ends which can never be attained, or toward the contemplation of that which was the fictitious image of an overwrought imagination. Could a student, eagerly pursuing his studies, and zealously reaching out for the veiled and hidden things of life, escape such contamination? Again, what were Hamlet's inherited tendencies? Surely no one, with a due knowledge of human nature, can assert that they were unblemished. Were there not particularly well marked mental characteristics of unwholesome kind in the lives of his mother and uncle? Is it assuming too much to suppose that in the death of the King of Denmark by his own brother, there is foreshadowed over the path of young Hamlet, an all-powerful force which he could not *will* to put away from him, and which attained its consummation in the death of the uncle at the hands of the nephew, or that the mother's want of moral stability may not have been transmitted in embryonic life, leaving its brand upon the mental condition of her offspring? After leaving his university at Wittenberg, at the age of thirty, where he had imbibed much of that habit of thought, no doubt, so current at that time, he lost his father. This was the first real blow of his life. A severe one to so affectionate a son; one sufficient to engender unhappy thought in a mind already weakened by inheritance and by surroundings. It weighed upon him constantly; an onus far too heavy for him to carry alone. It became an absorbing, dominant idea, resulting in a fondness for seclusion and for self-conscious, persistent contemplation. It was the first visible commencement in the work of mental disintegration. Then he is informed by the *ghost*, of the manner in which his father had died. This was the only stimulus needed to create that disruption of the ideational centres in respect to their anastomoses, for which the way had already been paved, and which resulted in that melancholic condition which ceased only with death. Is it not easy of comprehension, how an overwrought mind, congenitally unreliable, could be turned aside from a normal condition by a series of such life-long shocks? Notice, too, the manner in which such information is given! The ghost, first seen by Horatio and others, then by Hamlet, what was it but an ideal creation—a hallucination arising out of the overstrained cell in which had festered the dominant idea of the recently buried King; the residual force of the impression which had been formed during the life of the King, and which, acted on powerfully by the emotions, thus spent itself externally? See how the hallucination reappears when the necessary stimulus is applied. (Act III, Scenes III and IV.) The religious sentiment which withheld the suicidal desire of Hamlet, as well as the existence of the idea itself, are strong points in the chain of evidence, on which is based the assertion of his insanity, and of that division of it known as melancholia. In many cases of this disease, the religious fervor is the first manifestation of its outbreak, while in others there is often suicidal impulse. His first meeting with Ophelia is characterized by a great want of logical coherence and by bitter cynicism. He confesses to having nocturnal hallucinations (Act II, Scene III), and laments his own changed and saddened condition (Act II, Scene III). The celebrated soliloquy (Act III, Scene I,) is the morbidly conscious introspection of the Ego—the unhealthy tension of an all-dominant thought of which he cannot rid himself. The nature of his father's death is constantly before him; to it all else is referred. His insanity becomes manifestly evident to friends and relations. (Act I, Scene V. "He waxes desperate with imagination." Act II, Scenes I and III, Act III, Scene IV, *et al.*) He mistakes Polonius for another, and addresses him in a most erratic manner; and all his conversation is the emanation of the unhealthy, tyrannical and self-conscious idea of which he is possessed. What boots it that his fencing was the best in Denmark? One of the most accomplished chess-players that we have ever met, was a chronic case of insanity. He dies—and how? Poisoned. The faint reflection of an abnormal growth, which was mirrored in his earlier life, had grown to such dimensions as to assume the controlling power.

Thus passes away one, a victim, by inheritance and association, to a morbid psychological condition. Who will cast the stone at one thus dying, for the deeds he worked in the flesh, impelled by a nervous condition implanted in him which he could not conquer? Who, in instances of such a nature, can maintain that will is an innate, constant quantity, dependent upon nothing?

